# Transcriptional Regulation of a Bacteriophage Encoded Extracellular DNase (Spd-3) by Rgg in *Streptococcus pyogenes*

**DOI:** 10.1371/journal.pone.0061312

**Published:** 2013-04-17

**Authors:** Srivishnupriya Anbalagan, Michael S. Chaussee

**Affiliations:** Division of Basic Biomedical Sciences, The Sanford School of Medicine of the University of South Dakota, Vermillion, South Dakota, United States of America; University of Kansas Medical Center, United States of America

## Abstract

The *Streptococcus pyogenes* transcriptional regulator Rgg controls the expression of virulence-associated genes encoded both within the core genome and within horizontally transmissible DNA such as temperate bacteriophage. Previously, we showed that Rgg binds to the non-coding DNA upstream of the bacteriophage gene encoding an extracellular DNase Spd-3. In the current study, we further characterized Rgg-mediated regulation of *spd-3* expression. Two *spd-3* transcripts were identified by northern blotting. The 5′ ends were 27 and 594 nucleotides upstream of the start codon as determined with primer extension analysis and 5′ RACE (rapid amplification of c-DNA ends), respectively. Results obtained with gel shift assays showed that purified Rgg bound specifically to non-coding DNA containing the promoters of both transcripts. Transcriptional fusion analyses confirmed the presence of Rgg-repressible promoters within these DNA regions. In addition, repression was associated with direct DNA binding by Rgg as determined with chromatin immunoprecipitation (ChIP) coupled with quantitative PCR (qPCR). The results show that the chromosomally encoded transcriptional regulator, Rgg, directly represses both bacteriophage promoters controlling the expression of Spd-3. The results provide new information regarding the regulation of prophage encoded virulence factors of *S. pyogenes* and highlight the complex evolutionary history of *S. pyogenes* and temperate bacteriophage.

## Introduction

*Streptococcus pyogenes* causes several human diseases ranging in severity from self-limiting pharyngitis to life-threatening necrotizing fasciitis and streptococcal toxic shock syndrome [1]. The virulence of the pathogen varies temporally over the course of decades due to changes in both the pathogen and human immunity [2], [3]. The determination of the genome sequences of several isolates revealed a theoretically limitless *pan*-genome, or mobilome, comprised of mobile genetic elements (MGE) including bacteriophages, integrative conjugative elements, transposons, and insertion sequences [3]–[5]. MGEs significantly alter the composition of the chromosome. For example, between two and eight complete or partial bacteriophage genomes are present in the genomes of *S. pyogenes* and bacteriophage DNA can account for up to 12% of the chromosome [6], [7]. Often, the bacteriophage encode virulence factors including superantigens [8] and extracellular nucleases [9], [10], which profoundly influence interactions between the pathogen and its human host. Thus, chromosomal heterogeneity, including variation in the number and types of prophage within a chromosome, is responsible for much of the genetic diversity observed among clinical isolates and contributes to the clinical and temporal variation in the outcome of human colonization with *S. pyogene*s [7], [11]–[13].

*S. pyogenes* can produce up to four extracellular DNases [14], [15]. One (MF-1/DNaseB) is chromosomally encoded and is adjacent to *rgg* (also known as *ropB* [16]), which encodes a global transcriptional regulator [17]. The remainder are encoded by prophage. Therefore, the number of extracellular DNases potentially produced by an isolate varies depending on the prophage content of the chromosome.

Extracellular DNases have long been thought to be important in liquefying pus to promote bacterial dissemination. In addition, prophage-encoded extracellular DNase degrades bacterial DNA following induction of the lytic phase, which can further reduce viscosity at the site of colonization and promote transmission of the progeny virions to new bacterial hosts [9]. More recently, the prophage encoded extracellular nuclease Sda1 was found to degrade neutrophil extracellular traps (NETs) [18]. NETs are a component of innate immunity and are composed of a scaffold of neutrophil-derived chromatin and antimicrobial peptides, which entraps and kills microbes, including *S. pyogenes* [19]. Degradation of bacterial DNA also decreases macrophage mediated killing of *S. pyogenes* due to decreased stimulation of toll-like receptor 9, which recognizes unmethylated CpG-rich DNA [20]. Thus, extracellular DNase’s promote pathogen dissemination and survival, although some appear to be relatively more important than others [18], [21]. Importantly, the exoproteins contribute to virulence in both mouse models of invasive infection [20] and in a cynomolgus macaque model of pharyngitis [21].

The serotype M49 strain NZ131 possesses three prophages [22], including one consisting of only 16 kb that presumably has decayed. The remaining two prophages, NZ131.2 and NZ131.3, are 37,895 and 47,501 bp, respectively. NZ131.2 encodes a superantigen known as streptococcal pyrogenic exotoxin H (SpeH; [23]) and NZ131.3 encodes an extracellular nuclease known as Spd-3. Thus, strain NZ131 has two extracellular nucleases, the chromosomally encoded SdaB (MF-1) and the prophage encoded Spd-3.

Inactivation of the gene encoding the transcriptional regulator Rgg increased expression of both SdaB (Spy49_1692c; Mf-1) and Spd-3 (Spy49_1455) in the post-exponential phase of growth [17]. Subsequently, we found that Rgg binds to non-coding prophage DNA upstream of *spd-3* [24]. The purpose of the current study was to characterize further the role of Rgg in the regulation of the prophage encoded DNase Spd-3.

## Results

### Identification of *spd-3* Transcripts

**Figure 1:**
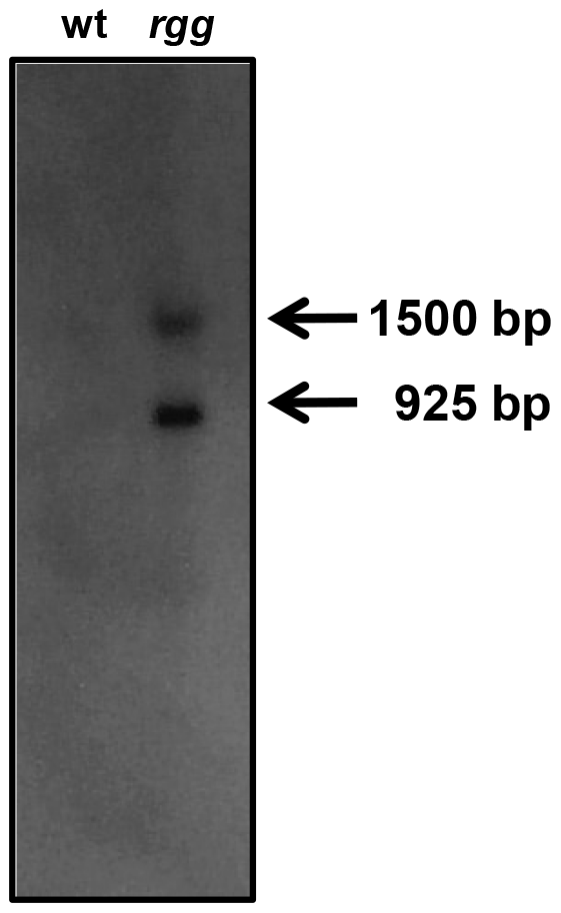
Detection of *spd-3* transcripts. Northern blotting using an *spd-3* specific probe and RNA isolated from the wild type (wt) and the *rgg* mutant (*rgg*) strains showed two transcripts in the sample obtained from the mutant strain. The approximate sizes were determined by using an RNA ladder (not shown).

As an initial step to characterize the regulation of *spd-3* expression, northern blotting was done using RNA isolated during the post-exponential phase of growth from both the wild-type *S. pyogenes* strain NZ131 and an *rgg* mutant. Two distinct transcripts were detected and both were more abundant in the mutant strain compared to the wild-type strain (Fig. 1), which was consistent with our previous finding that Rgg represses *spd-3* expression [17]. The more abundant transcript was approximately 925 bp in length and accounted for 65% of the transcript signal, as determined by densitometry.

### Mapping the *spd-3* Transcriptional Start Sites

**Figure 2:**
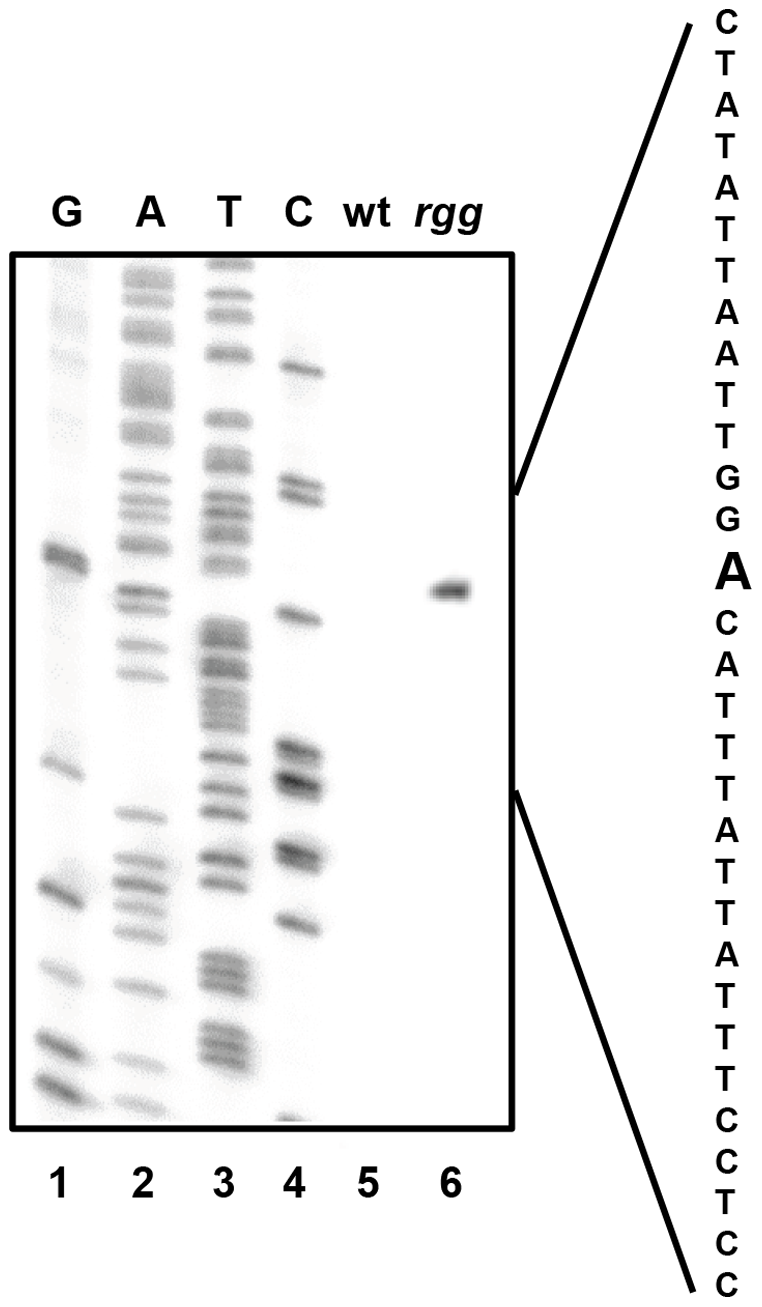
Primer extension analysis of *spd-3* transcripts. RNA isolated from the wt (lane 5) and *rgg* mutant strain (lane 6) was used to identify the 5′ end of the *spd-3* transcript. The DNA sequence (G A T C) generated from the non-coding DNA upstream of *spd-3* (lanes 1–4) is shown. The antisense strand DNA sequence proximal to the 5′ end is shown to the right and bold type indicates the start of the transcript.

**Figure 3:**
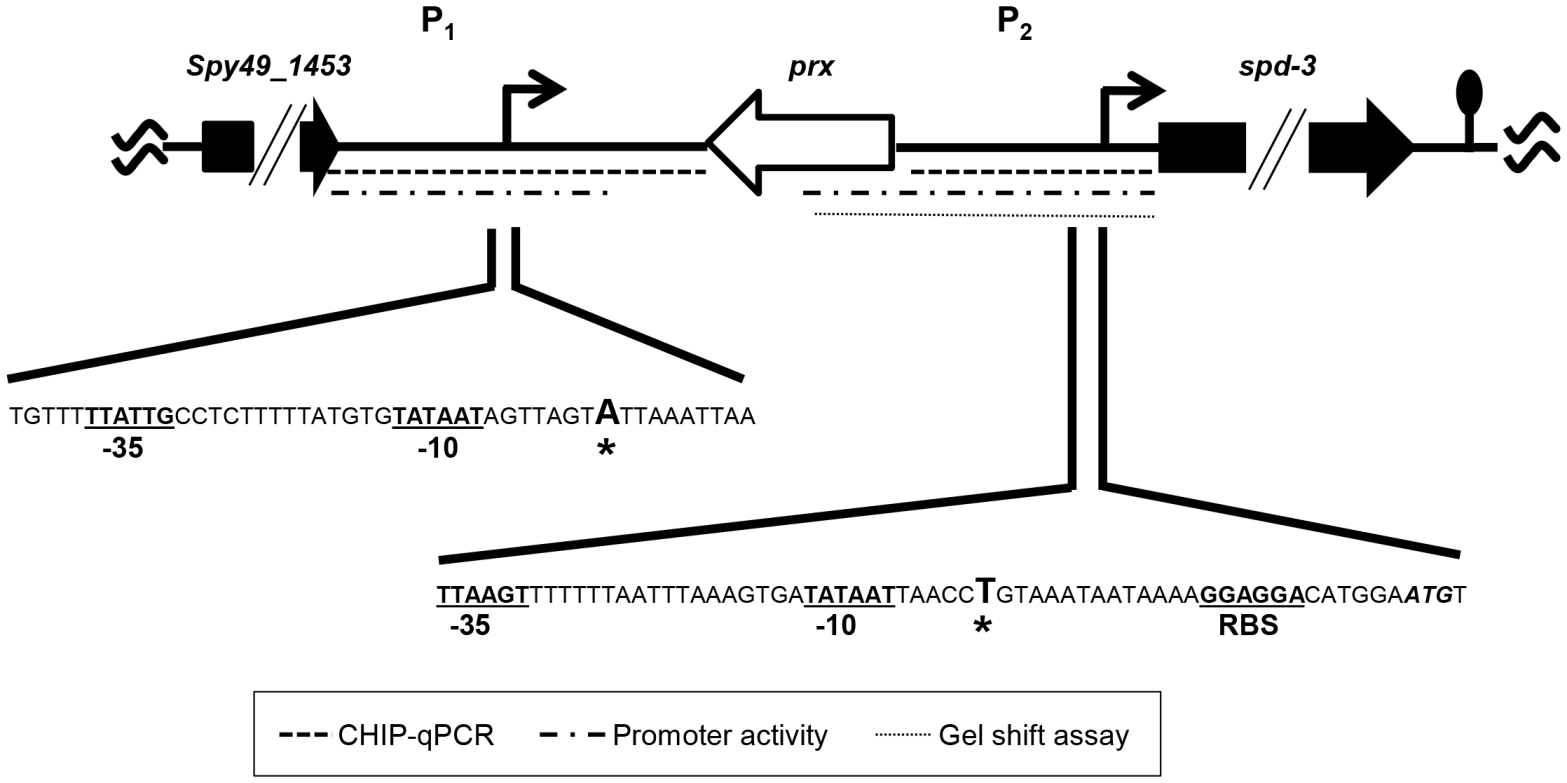
Schematic location of the two *spd-3* promoters. Transcriptional start sites are indicted by asterisks and bold face. The predicted -10 and -35 RNA polymerase binding sites of each promoter are indicated with bold type and underlined. A putative ribosome binding sites (RBS) associated with P_2_ is similarly indicated. The Spd-3 start codon is shown in italics and bold type. Dotted lines indicate the target DNAs used with ChIP-qPCR, gel-shft assays, and transcriptional fusion assays. The lollipop symbol indicates the location of a putative transcriptional terminator.

Primer extension analysis was used to determine the 5′ termini of the two transcripts. Extension with primer spd3PEc_96 (Table 1) showed a transcript that originated 27 bp upstream of the predicted *spd-3* open reading frame (ORF) (Fig. 2). This origin, coupled with a putative transcriptional terminator 91 bp downstream of the *spd-3* ORF, predicted a 919 bp transcript, which corresponded to the more abundant approximately 925 bp transcript detected by northern blotting (Fig. 1). A variety of primers were used in attempts to identify the start site of the longer transcript by using primer extension; however, we were unable to do so, possibly due to secondary structure formed within the large untranslated region. As an alternative approach, 5′ RACE was used and the results showed that the 5′ terminus was 594 bp upstream of the *spd-3* start codon (Fig 3). The results predicted a 1,487 bp transcript, which also correlated with the size of the larger transcript identified with northern blotting (Fig. 1). In addition, the start of transcription coincided with the non-coding DNA region previously shown to be bound by Rgg *in vivo* during the exponential phase of growth [24]. Analyses of the DNA proximal to the transcriptional start sites revealed the presence of putative -10 and -35 RNA polymerase binding sites (Fig. 3). The two transcriptional start sites were designated P_1_ and P_2_ (Fig. 3).

**Table 1:** Oligonucleotides.

Primer	Sequence^a^ (5′–3′)	Reference or source
spd3fwd	GCCAGACCCTTGCTGCTAATCCA	24
spd3rev	GGTGCCTGTAAAATACGAATAAATAAGT	24
spd3fwd1	GGCGTAGCATTTAAATAAACGGAA	This study
spd3rev1	GGCAAGGAGGGTAAAAATGCTAAC	This study
pspd3_IRfwd	GCGGATCCGTCGGACTAGTCTATGACAAA	24
pspd3_IRrev	GCCTCGAGATCCATGTCCTCCTTTTATTATTTAC	24
spd3_237fwd	TTTTACCCTCCTACTTATTTATTCG	This study
spd3_300_BamHIfwd	GCGGATCCCGGAATTAATTAAAATATTTTTGTCC	24
spd3_378_XhoIrev	GCCTCGAGAAGTAGCGGGAGAAGTGCTAATGG	This study
groEL2fwd	GCTACTCGACGTAACATTGTG	24
groEL2rev	GGAGCCTTCGTACCCAGCAT	24
spd3_cfwd	GCGGATCCATGTCTAAATCAAATCGTCGTA	This study
spd3_crev	GCCTCGAGTTCGGTTTCTAAATTACTATCTTC	This study
spd3_PEc_96	TCTGGCTGCCGTAACAGTACTTGTG	This study
spd3prx36	GAAGTAGCGGGAGAAGTGCTAATGG	This study
AAP-G	GGCCACGCGTCGACTAGTACGGGGGGGGGGG	This study
LucRev1	GCCAAGCTGGAATTCGAGCTCCCAT	This study
Nested prx	GGACAAAAATATTTTAATTAATTCCG	This study
AUAP	GGCCACGCGTCGACTAGTAC	This study

a Underlined nucleotides are restriction sites incorporated into primer.

### Rgg Binds Specifically to DNA Containing the P1 and P2 Promoters

**Figure 4:**
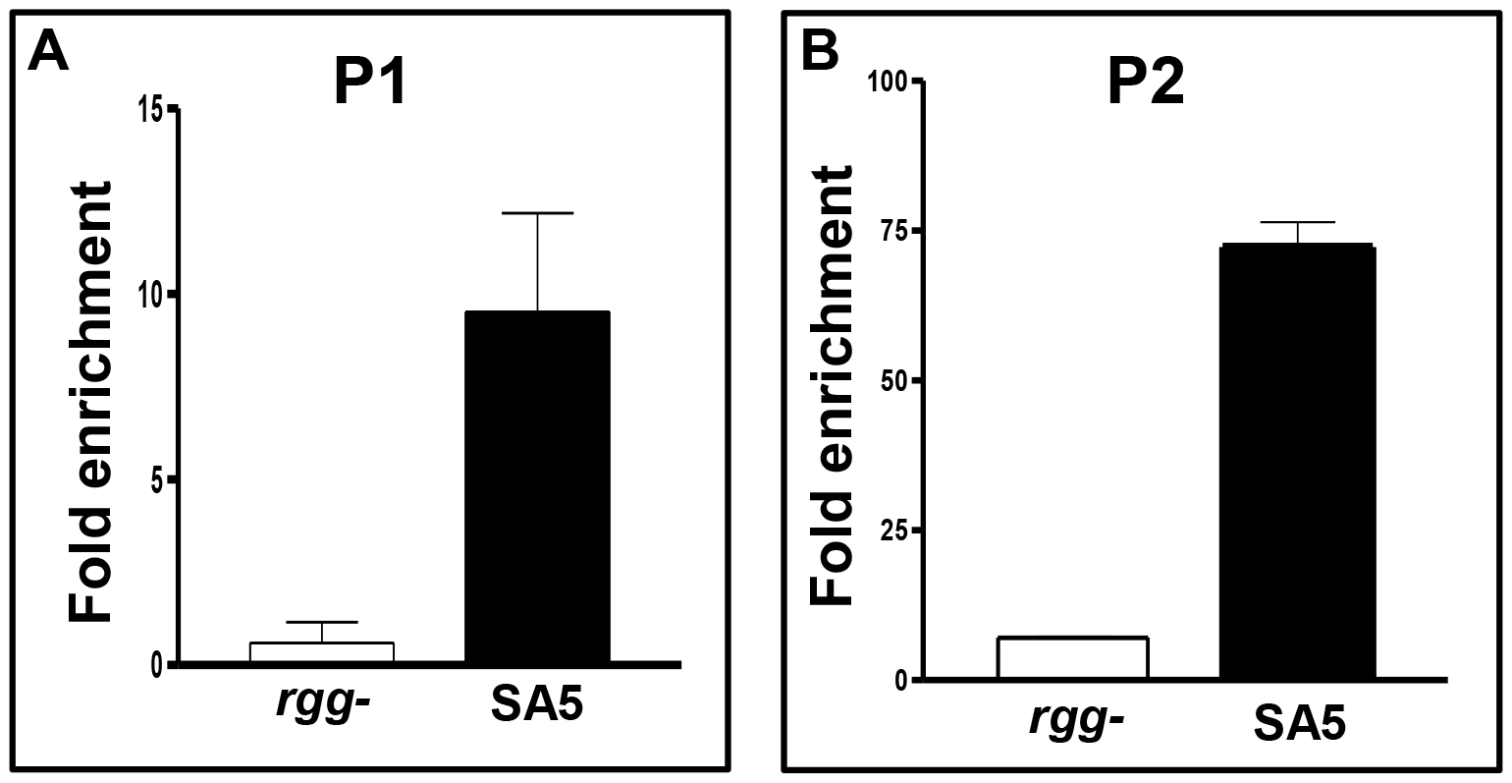
Rgg binds *in vivo* to *spd-3* promoter regions P1 and P2. ChIP and qPCR was used to measure the amount of P1 (Panel A) and P2 (Panel B) containing DNA bound by Rgg. Experiments were conducted at least three times, and the means and standard deviations are shown.

**Figure 5:**
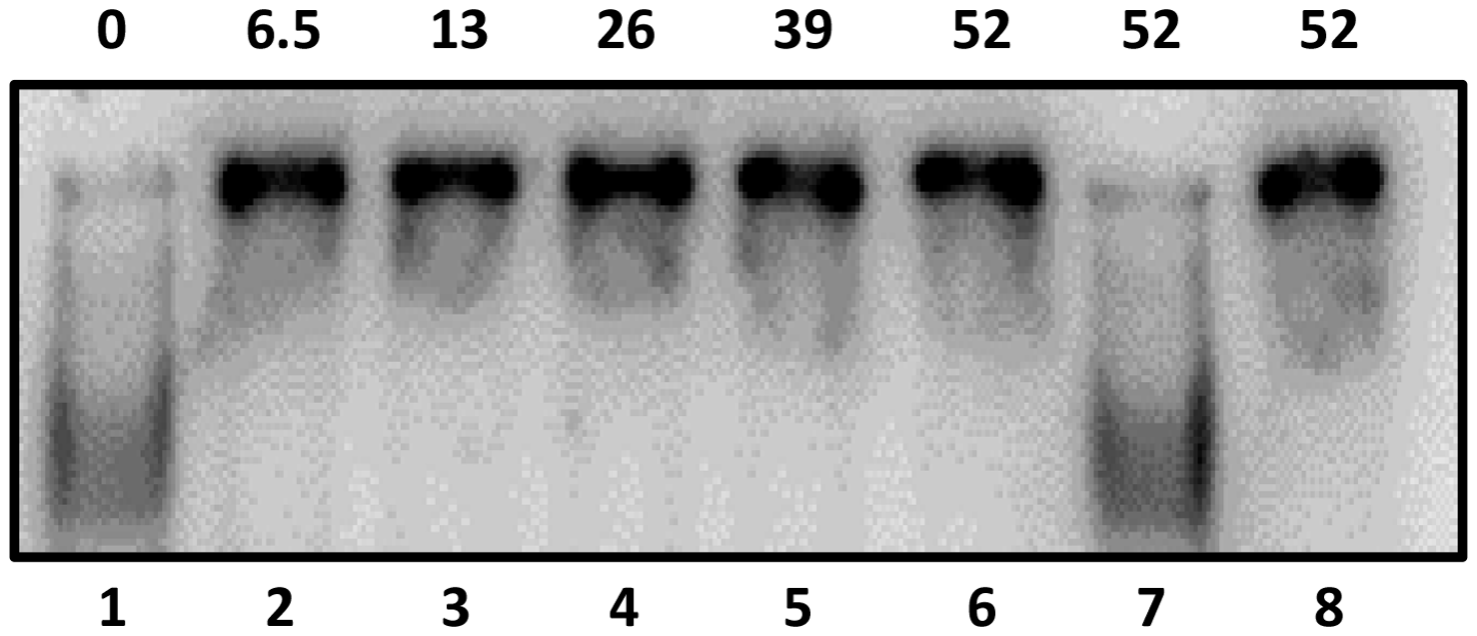
Rgg binds specifically to P2. Rgg binding to the non-coding DNA upstream of *spd-3* containing P2 was assessed by incubating with 0, 6.5, 26, 39 and 52 pmoles (Lanes 1–6) of purified Rgg with radiolabeled target DNA. Lane 7) Specific un-labeled competing DNA was added to the reaction. Lane 8) Labeled non-specific DNA (*groEL)* was added to the reaction.

ChIP coupled with DNA genechips (ChIP-chip) and gel-shift assays previously showed that Rgg binds to P_1_ [25]; however, we did not previously detect binding to P_2_. Therefore, we re-analyzed Rgg binding to both sites by using the more sensitive ChIP-qPCR procedure. As expected, DNA containing P_1_ was enriched by approximately 9-fold in strain SA5, which encodes an Rgg-Myc fusion protein used to facilitate immunoprecipitation, compared to the control strain (Fig. 4). In addition, the P_2_ region was enriched nearly 75-fold (Fig. 4). The results show that Rgg binds to both the P_1_ and P_2_ DNA (Fig. 4). Binding near P2 was further evaluated with gel-shift assays and the results confirmed that Rgg specifically binds to DNA containing P_2_ (Fig. 5).

### Rgg Directly Regulates *spd-3* Expression

To characterize the two *spd-3* promoters further, prophage DNA regions bound by Rgg (P1 and P2) were cloned adjacent to a promoterless firefly luciferase (*luc*) gene present in the shuttle plasmid pKSM720 [26]. The recombinant plasmids were used to transform both the wild-type and the *rgg* mutant strains to determine if expression from P1 and P2 was regulated by Rgg. The use of the plasmid based reporter system also allowed us to measure promoter activity apart from the prophage, thus avoiding confounding factors potentially associated with prophage induction, such as variation in gene copy number. Promoter activity was detected with P_2_ DNA and the presence of Rgg in the wild-type strain decreased transcription, consistent with other data indicating that Rgg represses *spd-3* transcription (Table 2). Similarly, a fragment containing both *spd-3* promoters (P1 and P2), showed significantly more activity compared to the fragment containing only P2 (Table 2). Again, more promoter activity was detected in the *rgg* mutant strain, consistent with Rgg-dependent repression of *spd-3* expression by direct binding to the promoter regions (Table 2).

**Table 2:** Both *spd-3* promoters (P1 and P2) are repressed by Rgg.

Promoter	[Luciferase] units	
	wild-type	*rgg* mutant
P2^a^	145 (69)	35,857 (20,462)
P1 & P2^a^	1,486 (855)	335,979 (61,184)

a A 457 bp fragment containing the P2 promoter or a 761 bp fragment containing both the P1 and P2 promoters was cloned adjacent to the luciferase reporter gene. The mean (standard deviation) from independent experiments is shown.

### Rgg Acts in *cis* to regulate *spd-3* Promoters

**Figure 6:**
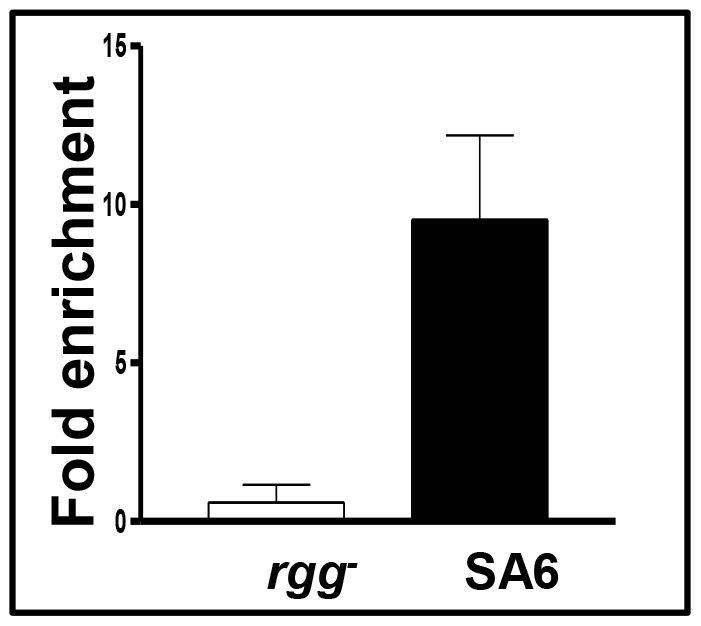
Rgg binds to episomal P2 containing DNA. The amount of the non-coding DNA upstream of *spd-3* cloned into a transcriptional reporter fusion plasmid was measured by quantitative PCR, as a negative control, *groEL* in ChIP samples obtained from the *rgg* mutant and strain SA6. Experiments were conducted at least three times, and the means and standard deviations are shown.

Finally, we used the transcriptional reporter system and ChiP-qPCR to determine if Rgg bound in *cis* to regulate expression of the *luc* gene. One PCR primer was specific to *luc* and a second was upstream of P_1_. The results showed that Rgg bound directly to the plasmid DNA to repress expression of *luc* (Fig. 6).

## Discussion

Temperate bacteriophage can ferry virulence-associated genes encoding a variety of toxigenic and enzymatic exoproteins among bacteria. In *S. pyogenes*, prophages encode superantigens, which are responsible for toxic shock syndrome; extracellular DNases, which assist in the pathogen’s escape from the innate immune response; and surface exposed proteins, which have not been characterized. Despite the importance of the gene products to human diseases caused by *S. pyogenes*, comparatively little is known about the regulation of their expression. Here, we examined the role of Rgg in the repression of the bacteriophage encoded extracellular DNase Spd-3. Two *spd-3* transcripts were identified and the 5′ termini determined. The abundance of both transcripts was elevated in an *rgg* mutant strain and Rgg bound to DNA containing the promoters of both transcripts, indicating that Rgg binding represses expression. To analyze regulation outside the context of the prophage, which can excise from the chromosome and thereby increase *spd-3* copy number, a plasmid-based reporter system was used to measure *spd-3* promoter activity. The results confirmed that Rgg repressed expression from both promoters. Finally we showed with ChIP-qPCR that Rgg bound to DNA in *cis* to effect transcription of the reporter gene. The results show that the chromosomally encoded regulator Rgg represses expression of the prophage-encoded virulence gene *spd-3* expression by direct binding to prophage promoters.

Rgg regulates dozens of genes in strain NZ131 in a growth-phase associated manner, including many known to contribute to virulence [16], [27], [28]. It does so, in part, by changing its DNA binding specificity in response to glycolytic flux via direct protein-protein interaction with LacD.1 [29]. In addition, Rgg binds to hydrophobic peptides, which modulates regulatory activity [30], [31]. The genes controlled by Rgg are functionally diverse and include both cell-associated proteins and soluble exoproteins associated with virulence [17], [25]. For example, Rgg represses expression of the cell wall-associated antiphagocytic M protein in the exponential phase of growth and activates expression of a secreted cysteine protease SpeB in the post-exponential phase [16], [27], [32]. The results of the current study, together with results from previous studies, show that Rgg coordinates the expression of both prophage and chromosomally-encoded virulence genes in response to environmental cues. Consistent with these findings, Rgg contributes to the outcome of *S. pyogenes* infection as assessed with animal models and as observed in human epidemics of invasive disease [33] [34].

Excision of prophage from the chromosome is associated with increased gene copy number and often with simultaneous expression of prophage-encoded virulence genes. In some instances the repressor of the lytic phase also directly represses expression the prophage-encoded virulence genes. For example, expression of the prophage-encoded Shiga toxin (StxAB) in *Escherichia coli* is primarily controlled by a prophage repressor that also controls lysogeny [35], [36]; thus *stxAB* expression is dependent on prophage induction. In contrast, expression of the prophage-encoded cholera toxin (Ctx) is controlled by several chromosomally encoded regulators and is not dependent on induction of the encoding prophage [37]. Thus two paradigms have emerged from the study of the regulation of prophage encoded virulence factors.

In *S. pyogenes*, expression of prophage encoded virulence genes, including those encoding superantigens, has been associated with the induction of prophage [4], [8], [9]; however, induction does not appear to be necessary for expression [4]. Moreover, expression of the phage-encoded extracellular DNase Sdn (SpyM3_1409) decreases following mitomycin C induction of prophage, despite an increase in *sdn* copy number [4]. In this study, we showed that Rgg directly represses the promoters controlling *spd-3* expression, even in the absence of prophage induction, as determined by using a plasmid based reporter system. Although our investigation focused on the regulation of *spd-3* expression, the results are consistent with a model in which the expression of prophage encoded virulence factors in *S. pyogenes* is not dependent on induction the lytic cycle and is controlled, at least in part, by chromosomally encoded regulatory loci. Given the relevance of prophage encoded superantigens and secreted DNases to human disease, additional investigation into the regulation of other prophage-encoded virulence factors is warranted.

Phenotypic variation is a hallmark of many pathogens, including *S. pyogenes*, and can result in heterogeneous clinical outcomes of infection. We previously showed that Rgg binds to non-coding DNA upstream of a prophage integrase/excisionase (Spy49_0746c) to repress expression [17], [24]. Increased expression of the integrase/exicisionase in an *rgg* mutant strain was associated with a decrease in the frequency of prophage excision from the chromosome [17]. The current study extends the idea that Rgg regulates specific prophage encoded genes, which alters the phenotype of the pathogen. Given the tremendous variation in the number and composition of bacteriophage among different isolates of *S. pyogenes* [7], we speculate that the direct regulation of MGEs by Rgg contributes, directly or indirectly, to the variation in the Rgg regulon observed among various isolates of *S. pyogenes* [38] and potentially the clinical outcome of human infection.

## Materials and Methods

### Bacterial Strains and Culture Conditions

*S. pyogenes* strain NZ131 (serotype M49) was isolated from a patient with acute post-streptococcal glomerulonephritis (Table 3) [22]. NZ131 and its genetic derivatives including *rgg*- (32) and SA5 and have been previously described (24). *S. pyogenes* strains were grown with Todd-Hewitt broth (Becton Dickinson, Spark, MD) containing 0.2% (wt/vol) yeast extract at 37°C in a 5% CO_2_ atmosphere without agitation. *E. coli* DH5α was grown with Luria-Bertani medium at 37°C with agitation. When necessary, antibiotics was added to the growth media at the following concentrations: carbenicillin at 100 µg/ml for *E. coli*; spectinomycin at 100 µg/ml for both *E. coli* and *S. pyogenes*; erythromycin at 2.5 µg/ml for *S. pyogenes*; kanamycin at 50 µg/ml for *E. coli* and 500 µg/ml for *S. pyogenes*.

**Table 3:** Bacterial strains and plasmids.

Strain or plasmid	Description	Source or reference
**Strains**		
***E. coli***		
DH5α	*hsdR17 recA1 gyrA endA1 relA1*	Invitrogen
***S. pyogenes***		
NZ131	M49 serotype	D.R. Martin, New Zealand
*rgg-*	NZ131 *rgg* mutant, Em^R^	32
* SA5*	NZ131*rgg^-^* complemented with pSA3, Em^R^, Kan^R^	24
*SA6*	*SA5* transformed with pSA12	This study
* *wt::*luc*	NZ131 transformed with pKSM720, Spec^R^	24
wt::P*spd3-luc*	NZ131 transformed with pSA6, Spec^R^	24
wt::P*spd3-379luc*	NZ131 transformed with pSA27, Spec^R^	This study
wt::P*spd3-457luc*	NZ131 transformed with pSA28, Spec^R^	This study
* rgg-*::*luc*	NZ131*rgg* mutant transformed with pKSM720, Spec^R^	24
* rgg-*:: P*spd3-luc*	NZ131 *rgg* mutant transformed with pSA12, Spec^R^	24
*rgg-*::P*spd3-379luc*	NZ131 transformed with pSA27, Spec^R^	This study
*rgg-*::P*spd3-457luc*	NZ131 transformed with pSA28, Spec^R^	This study
**Plasmids**		
pGEM-T-Easy		
pKSM720	GAS replicating plasmid with firefly luciferase and RBS, Spec^R^	26
pSA12	Non-coding region upstream of *spd-3* was cloned into pKSM720, Spec^R^	24
pSA27	Non-coding region between -761 and -382 upstream of *spd-3* was clonedinto pKSM720, Spec^R^	This study
pSA28	Non-coding region between -1 and -457 upstream of *spd-3* was clonedinto pKSM720, Spec^R^	This study
pSA29	Non-coding region between -225 and -472 upstream of *spd-3* was clonedinto pGEM-T-easy vector, Amp^R^	This study

### DNA Manipulation

To isolate plasmid DNA from *E. coli*, either the QIAprep Spin Miniprep Kit (Qiagen, Valencia, CA) or Maxi/Midi prep purification systems (Qiagen) was used. DNA fragments were PCR amplified with GoTaq DNA polymerase (Promega, Madison, WI) and the amplified DNA was separated by using agarose gel electrophoresis and purified using the SpinPrep Gel DNA kit (EMD Milliopore, Darmstadt, Germany). DNA sequencing to confirm various constructs was done at Iowa State University (Ames, IA).

### Promoter Activity Assays

A shuttle plasmid (pKSM720) containing *luc* [26], which encodes firefly luciferase, was used to construct transcriptional fusions. Two DNA regions upstream of *spd-3* (−761 to −382 and −1 to −237) were amplified using pspd3_IRfwd and spd3_378_XhoIrev; spd3_237fwd and pspd3_IRrev primer combinations, respectively (Table 1). The 379 bp and 237 bp DNA fragments were gel purified, digested with *BamH*I and *Xho*I, and cloned 5′ to *luc* between *Bgl*II and *Xho*I of pKSM720 (Table 3) to create pSA27 and pSA28, respectively (Table 3). NZ131 was transformed with pKSM720, pSA27, and pSA28 by electroporation to create wt::*luc*, wt::P*spd3-379luc*, and wt::P*spd3-*457*luc*, respectively (Table 3). Similarly, the NZ131 *rgg* mutant was transformed with pKSM720, pSA27, and pSA28 to create *rgg^-^*::*luc*, *rgg-*::P*spd3-379luc*, and *rgg^-^*::P*spd3-457luc*, respectively (Table 3). Construction of the recombinant plasmids was confirmed by PCR. The *S. pyogenes* strains containing the transcriptional fusion plasmids were grown with THY broth to the exponential phase of growth (*A*_600_∼0.35) and luciferase activity was measured according to manufacturer’s instructions (Promega).

### Electrophoretic Mobility Shift Assays (EMSA)

An Rgg-maltose binding protein fusion protein (Rgg-MBP) was expressed in *E. coli* and purified as previously described [24]. Non-coding DNA upstream of *spd-3* (corresponding to nucleotides −1 to −457 bp relative to the *spd-3* start codon) was amplified by using primers spd3fwd1 and spd3rev1 primers and NZ131 genomic DNA as a template (Table 1). The fragment was isolated by using agarose gel electrophoresis, purified, and cloned into pGEM-T-easy (Promega, Madison, WI) to create pSA29. As a non-specific control of DNA binding, a similarly sized fragment was similarly prepared by using groEL2fwd and groEL2rev primers, which are specific to the *groEL* ORF (Table 1). The fragments were excised from pGEM-T easy, gel purified, dephosphorylated, and end labeled with [γ^32^P] ATP using polynucleotide kinase. Different amounts of Rgg-MBP were incubated in 25 µl of binding buffer (25 mM Tris-Cl, pH 7.5, 0.1 mM EDTA, 75 mM NaCl, 1 mM dithiothreitol, 10% glycerol, and 0.5 µg/ml of calf thymus DNA) at room temperature for 20 min. Competition experiments were conducted by including unlabeled DNA prior to protein addition. The reaction mixtures were separated with a 6% nondenaturing polyacrylamide gel. The gels were dried, exposed to an Amersham Biosciences storage phosphor screen and imaged with a Typhoon 9400 instrument (GE Healthcare, Piscataway, N.J.).

### RNA Isolation and Northern Blotting

Overnight cultures of NZ131 and the *rgg* mutant were inoculated into 40-ml THY broth to an *A*_600_ of 0.08. The cultures were grown to the post-exponential phase of growth (*A*_600_ ∼ 0.6). Total RNA was isolated as described previously [17]. The concentration and quality of RNA was assessed with an Agilent 2100 Bioanalyzer (Agilent, Palo Alto, CA) using an RNA 6000 Nano LabChip kit (Agilent). Fifteen micrograms of total RNA from each strain was separated with a 1.5% agarose-0.66 M formaldehyde gel in morpholinepropanesulfonic acid (MOPS) running buffer (20 mM MOPS, 10 mM sodium acetate, 2 mM EDTA; pH 7.0). RNA was blotted onto Hybond N^+^ membranes (GE Healthcare Biosciences, Pittsburgh, PA.) with the Turboblotter alkaline transfer system (Schleicher & Schuell, Keene, N.H.), according to the manufacturer’s instructions. RNA was fixed to the membrane by baking at 80°C for 30 min. The *spd-3* coding DNA was amplified using spd3_cfwd and spd3_crev primers (Table 1), separated by agarose gel electrophoresis, the fragment excised from the gel, and purified as described above. Using the purified *spd-3* coding DNA as a template, [α-^32^P]dCTP radiolabelled probes were synthesized by the random-primed method (Ready-To-Go Labeling Kit; Pharmacia). Membranes were hybridized under aqueous conditions at 65°C with the radiolabelled probes. The blots were washed and exposed to an Amersham Biosciences storage phosphor screen and imaged with a Typhoon instrument.

### Primer Extension Analysis

The 5′ end of the *spd-3* transcript was determined with 15 µg of RNA isolated from NZ131 and the *rgg* mutant using an AMV reverse transcriptase primer extension kit (Promega) according to manufacturer’s instructions. The spd3PEc_96 primer (Table 1) was end-labeled with [γ^32^P] ATP using polynucleotide kinase and the extension products were separated with a 6% polyacrylamide-urea sequencing gel. The 5′ end was mapped by comparison to a sequencing reaction generated with a SequiTherm EXCEL™ II DNA Sequencing Kit (Epicentre Biotechnologes, Madison, Wisconsin) with the end-labeled spd3PEc_96 and a DNA template that contained the entire non-coding DNA upstream of *spd-3*.

### 5′ Rapid Amplification of c-DNA Ends (5′-RACE)

The 5′ end of the larger *spd-3* transcript was determined using the 5′ RACE System for Rapid Amplification of cDNA Ends, Version 2.0 (Invitrogen, Carlsbad, California) according to manufacturer’s instructions. First strand cDNA was synthesized using 15 µg of total RNA, the gene specific primer spd3prx36, and Superscript III Reverse Transcriptase (Invitrogen). After the first strand cDNA synthesis, the mRNA template was removed with the RNase cocktail (Ambion, Austin, Texas). Single stranded cDNA was purified using a DNA Clean and Concentrator-5 kit (Zymo Research Irvine, California). A homopolymeric tail was added to the 3′-end of the cDNA using recombinant TdT (Invitrogen) and dCTP. Five µL of dC-tailed single stranded c-DNA was amplified using abridged anchor primer (AAP-G) and nested prx primers (Table 1). The amplified product was diluted 100-fold and 5 µL was used in a second-round of PCR amplification with abridged universal amplification primer (AUAP) and nested prx primers. The PCR products were separated by electrophoresis on a 1% agarose gel, purified, and sequenced at the Iowa State Sequencing facility.

### ChIP-qPCR

A ChIP assay was performed using the experimental protocol previously described [24]. Briefly, cultures of *S. pyogenes* strains *rgg^-^* [32] and SA5 (encoding an Rgg-myc fusion protein [24]) were grown to an *A*_600_ of approximately 0.6, which corresponds to the post-exponential phase of growth. The cultures were treated with 1% formaldehyde (w/v). DNA bound to Rgg-Myc was immunoprecipitated with a monoclonal antibody to Myc (Invitrogen, Carlsbad, CA). Control samples were similarly prepared from an NZ131 *rgg* mutant strain. Immunoprecipitated DNA was purified and specific regions quantitated with PCR using ABsolute SYBR Green ROX Mix (ABgene House, Surrey, United Kingdom). Primer pairs pspd3_IRfwd, spd3_378_XhoIrev and Spd3_237fwd, pspd3_IRrev were used to quantitate DNA containing P1 and P2, respectively (Table 2). Primers pspd3_IRfwd and LucRev1 were used to measure DNA cloned upstream of the *luc* gene (Table 2). For the control region, primers groEL2fwd and groEL2rev were used (Table 2). Enrichment was normalized to the amount of non-specific *groEL* DNA in precipitated samples, as previously described [24].
